# An Assessment of Periodontal Status and Oral Hygiene in Patients with Hypertension in the South-West Region of Romania

**DOI:** 10.3390/biomedicines13030696

**Published:** 2025-03-12

**Authors:** Ana Pavalan, Allma Roxana Pitru, Dorin-Nicolae Gheorghe, Cristina Florescu, Dora Maria Popescu, Ana Maria Rîcă, Flavia Nicolae, Adina Andreea Turcu, Petra Surlin

**Affiliations:** 1 Department of Periodontology, Research Center of Periodontal-Systemic Interactions, Faculty of Dental Medicine, University of Medicine and Pharmacy of Craiova, 200349 Craiova, Romaniapopescu131@yahoo.com (D.M.P.);; 2 Department of Oral Pathology, Faculty of Dental Medicine, University of Medicine and Pharmacy of Craiova, 200349 Craiova, Romania; 3 Department of Internal Medicine and Cardiology, Faculty of Medicine, University of Medicine and Pharmacy of Craiova, 200349 Craiova, Romania; 4 Department of Odontology, Faculty of Dental Medicine, University of Medicine and Pharmacy of Craiova, 200349 Craiova, Romania; 5 Department of Oro-Dental Prevention, Faculty of Dental Medicine, University of Medicine and Pharmacy of Craiova, 200349 Craiova, Romania

**Keywords:** periodontal status, periodontal index, hypertension, oral hygiene, dental implants, cardiovascular disease

## Abstract

**Background:** This cross-sectional analytical study evaluates oral hygiene and periodontal status in patients with hypertension, given the established link between cardiovascular diseases and poor periodontal health. **Methods:** A total of 24 hypertensive patients (15 male; 9 female) and 30 healthy controls (19 male; 11 female) from Craiova, Romania, were assessed using Periodontal Probing Depth (PPD), the Plaque Index (PLQ), and the Bleeding on Probing Index (BPI). **Results:** The mean age was 62.2 years in the hypertensive group and 47.4 years in the control group. Oral hygiene was poorer in hypertensive patients (PLQ: 73% vs. 24.1% in controls), with higher PPD (5.2 mm vs. 3.7 mm) and BPI (82% vs. 23%). Among patients with dental implants, PLQ was 24% in hypertensive individuals vs. 15.6% in controls, PPD was 4.4 mm vs. 2.9 mm, and BPI was 44% vs. 15.5%. **Conclusions:** These findings indicate a higher risk of periodontal disease in hypertensive patients, though dental implants may decrease some adverse effects by improving oral hygiene and periodontal health in this population. In conclusion, our study highlights that patients with high blood pressure have a less favorable periodontal status compared to healthy controls.

## 1. Introduction

As described by the “Periodontal Medicine” concept and broadly acknowledged by experts in both fields, periodontal disease (PD) and cardiovascular diseases (CVDs) are linked by common pathogenic mechanisms on multiple levels, significantly contributing to the development of integrated preventive and therapeutical approaches [1,2]. Such major public health issues are mainly chronic, considerably adding to the global burden of long-term diseases that require constant medical supervision and care [3,4].

PD is caused by the subgingival accumulation of bacterial plaque or biofilm that triggers a low-grade chronic inflammation of the periodontal tissues that support the teeth [5]. Host-upregulated pro-inflammatory cytokines mediate and fuel this inflammatory reaction [6]. The initial stage of the inflammation is set within the superficial layer of the periodontal tissues, the gingiva. Gingivitis is usually characterized by bleeding gums, which increase in volume and have a red appearance [7]. If no treatment is administered, the inflammation will progress towards the deeper periodontal structures and eventually to the alveolar bone, leading to the formation of periodontal pockets and bone resorption. Eventually, the teeth will gain increasing mobility and require extraction [8]. According to the 2018 classification of PD, periodontitis is characterized by three key elements: loss of clinical attachment by the formation of periodontal pockets (clinical attachment loss/level = CAL), radiographic alveolar bone loss (RBL), and signs of gingival inflammation (bleeding upon probing) [1].

Cardiovascular diseases include several types of heart or circulatory-linked conditions such as elevated blood pressure, hypertension, coronary artery disease, or heart attack [9]. The main mechanism for the onset of CVDs is the accumulation of lipid deposits inside blood vessels (atherosclerosis) that gradually narrow their lumen and eventually prohibit efficient blood circulation [9]. Several risk factors add to this main mechanism, accelerating the process due to smoking, lack of physical activity, obesity, and hereditary background [10,11].

Linking PD and CVD has offered the scientific background necessary to acknowledge that patients suffering from periodontal inflammation also exhibit a higher risk of CVD onset, as supported by the findings of epidemiological studies on the topic [12,13]. The main pathogenic mechanism put forward and commonly accepted for this connection is the systemic dissemination of periodontal bacteria and pro-inflammatory cytokines from the periodontium to the general blood circulation system [14]. This kind of event can occur when the increased blood flow inside the inflamed gingiva is damaged by mechanical intervention such as tooth brushing, chewing, or even dental procedures such as scaling, subgingival instrumentation of periodontal pockets, or periodontal surgery performed on inflamed tissue [15]. Reaching the blood flow, the bacteria can graft on the arterial wall and induce structural damage to the endothelial lining and subsequent endothelial dysfunction and formation of atheromatous plaque deposits [14,15]. These events create the setting of potential life-threating incidents in the future. Alongside the bacterial involvement in the PD and CVD association, it has also been shown that pro-inflammatory cytokines, such as interleukin-1 (IL-1), IL-6, C-reactive protein (CRP), and tumor necrosis factor-alpha (TNF-α), can modify blood circulation [16,17]. During periodontal inflammation or other bacterial-caused dento-maxillary changes, the levels of these cytokines are usually upregulated, and if entering the systemic circulation, they can stimulate atherosclerosis [15,17,18].

The oral health status of CVD patients often exhibits symptoms, such as xerostomia, that consequently contribute to an increased risk of dental caries and gingival inflammation [19]. The reduced salivary flow in CVD patients is a common consequence of their medication, which is mainly used for anti-hypertensive effects [20]. Other medications used in CVD patients, such as anticoagulants, can increase the complexity of specific dental procedures, mainly oral surgery, due to an increased bleeding risk [21]. Especially in the case of treating periodontal conditions in CVD patients, a close collaboration between specialists is required. Periodontal procedures, including scaling and root planning, antibacterial therapy, and patient motivation and instruction on keeping good oral health practices, are essential for reducing inflammation, both locally and systemically [22]. Adjunctive therapies such as the use of chlorhexidine-based mouth washes and subgingival rinses are recommended for a limited time period [22]. Conversely, in patients with CVD, cardiologists and general practitioners should indicate oral and periodontal screening and subsequent required treatment [23].

In recent decades, dental implant therapy has increased in popularity and prevalence among patients seeking to improve their quality of life and health. While providing improved aesthetics and function, dental implants offer a viable long-term solution for an increasingly wider range of patients, including those suffering from CVDs [24]. Nevertheless, in these patients, a thorough assessment of their cardiovascular conditions and control is required before implant surgery to reduce the risk of complications [24,25,26]. Dental implants can also improve the periodontal status by stabilizing prosthetic teeth. Thus, the mechanical load on remnant natural teeth is reduced and, most importantly, the oral hygiene habits of patients are improved, which is mandatory for the long-term efficiency of the implant therapy [27,28].

We set out to assess oral hygiene and periodontal status in patients with hypertension and to compare the recorded results to a control group of healthy patients. Furthermore, we sought to evaluate if hypertension patients with dental implants exhibited a different status than non-implanted ones.

## 2. Materials and Methods

### 2.1. Study Design

This cross-sectional analytical study was designed to evaluate the difference in periodontal status for patients with hypertension and the potential impact of dental implant therapy. The study was conducted in the cardiology clinic of the Craiova Clinical Municipal “Filantropia” Hospital and the University of Medicine and Pharmacy of Craiova, an administrative center of South-West Romania, on patients originating from the region.

**Figure 1:**
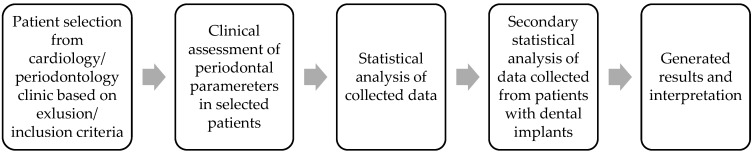
Study design flowchart.

The study protocol was approved by the Ethics Committee of the University of Medicine and Pharmacy of Craiova (no. 214/08.12.2021) and of the Clinical Municipal “Filantropia” Hospital of Craiova, Romania (no. 1606/26.01.2022). Informed consent was obtained from all participants prior to their inclusion in the study. The study adhered to the principles outlined in the Declaration of Helsinki for medical research involving human subjects. The sample size was estimated considering the addressability rate of patients to the involved clinics averaging in a 6-month interval (from March to September 2022) and the required inclusion and exclusion criteria (Figure 1).

### 2.2. Patient Selection

The study included 54 patients divided into two groups:

- Test Group: 24 patients with high blood pressure (15 males and 9 females with systolic blood pressure (SBP) of 130 mmHg or higher or a diastolic blood pressure (DBP) of 80 mmHg or higher [29]) and no previously diagnosed periodontal conditions.
- Control Group: 30 patients (19 males and 11 females) with no previously recorded cardiovascular or periodontal diagnosis.

Selected patients were aged between 45 and 75 years. For the test group, we selected patients presenting in the cardiology clinic diagnosed with hypertension and referred to the periodontology clinic for status evaluation. We chose those with no history of cardiovascular disease and periodontal pathology for the control group. The exclusion criteria envisaged pregnancy or lactation, diabetes or other systemic diseases affecting periodontal status, recent antibiotic or anti-inflammatory therapy (within 3 months), and smoking or tobacco use.

The educational status of the patients was evaluated by taking into consideration their level of education: high-school diploma or less (≤12 years formation); university diploma (12–15 years formation); post-graduate diploma (≥15 years formation) (Figure 1).

### 2.3. Periodontal Assessment

Periodontal status was evaluated by employing three primary parameters via a single and calibrated investigator.

1. Periodontal Probing Depth (PPD): This is measured at six sites per tooth (mesiobuccal, centrobuccal, distobuccal, mesiooral, centrooral, and distooral) using a standardized periodontal probe (UNC-15, Medesy, Maniago, Italy).
2. Plaque Index (PLQ): This is determined by assessing the presence of dental plaque on all tooth surfaces. The percentage of surfaces with plaque was recorded.
3. Bleeding on Probing Index (BPI): This is evaluated by gently probing the gingival sulcus at four sites per tooth (mesiobuccal, centrobuccal, distobuccal, and oral). The presence or absence of bleeding within 30 s was recorded.

### 2.4. Data Collection

For each patient, periodontal charts were filled with the information generated by the periodontal assessment (number of teeth and implants, PPD, PLQ, and BPI). These parameters, together with the number of existing teeth, were then expressed as mean values for each patient. The mean values and demographic/educational data were then aggregated in Excel (Microsoft, Irvine, CA, USA) and subjected to statistical analysis.

### 2.5. Statistical Analysis

The data recorded were analyzed using statistical software (GraphPad Prism 9.2.0, San Diego, CA, USA). The primary outcome measures were teeth number, PPD, PLQ, and BPI. We performed statistical comparisons between the test and control groups via independent *t*-tests to compare mean values of PPD, PLQ, and BPI between groups; ANOVAs were used to assess differences in periodontal parameters among subgroups (e.g., patients with and without implants). The power analysis of our results was performed using a post hoc Power Calculator (www.clincalc.com) at a power factor of 80% for each of the two groups, assuming an alpha level of 0.05. The results yielded an achieved power between 99% and 100% for the assessed parameters in patients with no dental implants and between 50.1% and 99.4% for patients with dental implants. We performed the Pearson correlation test for each group to analyze the educational and periodontal status of the patients (*p* < 0.05 for statistical significance).

## 3. Results

### 3.1. Demographic Data

The mean age within the test group was 62.2 years (SD: ±3.17; range 50–75 years). For the control group, the mean age was 47.4 years (SD: ±3.82; range 45–50 years). Both groups were similar in terms of gender distribution and geographic location, ensuring comparability. The test and control groups consisted of patients with natural teeth only and patients with natural teeth and dental implants (Table 1).

In both groups, the participants with high-school diplomas were the most prevalent (16 in the test group, 66.6%; 21 in the control group, 70%), while the rest of the participants had university degrees (8 in the test group, 33.3%; 9 in the control group, 30%). This exhibited a balanced distribution of the patients in the groups based on their educational status.

The patients from both groups with dental implants originated from both rural (40% of patients) and urban areas (60% of patients) and had high-school (60% of patients) diplomas as well as university (40% of patients) degrees, exhibiting a balanced distribution.

The sociodemographic characteristics of the participants were balanced among all analyzed parameters, ensuring the comparability of the clinical results.

### 3.2. Periodontal Status Parameters

**Figure 2:**
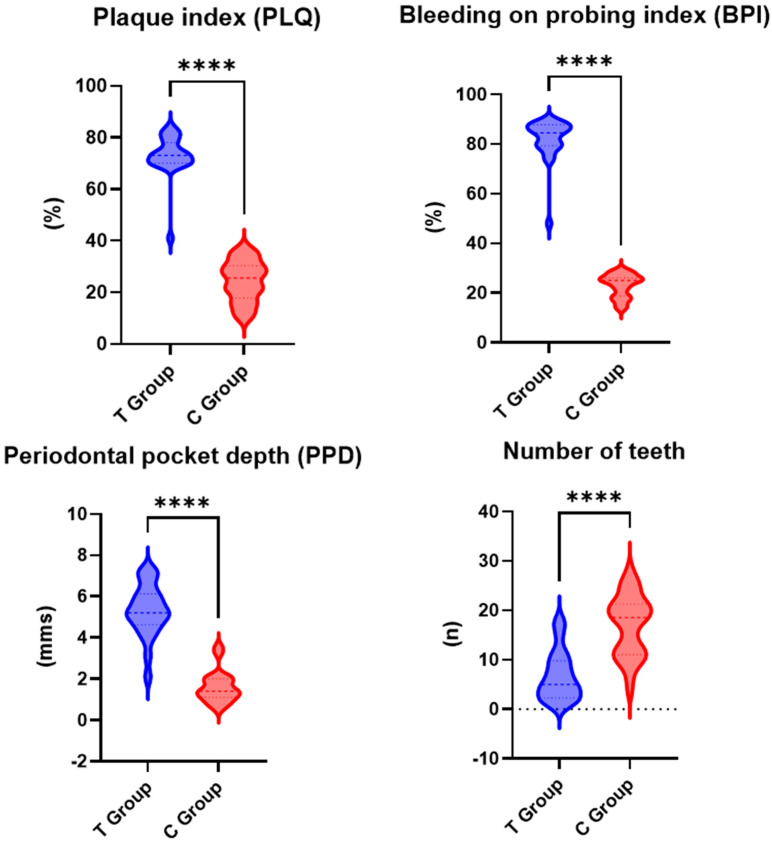
Periodontal parameters for test group (T) and control group (C); ****—*p* < 0.001.

All the assessed periodontal parameters exhibited higher values for the test group than the control group, with the differences between the two groups being statistically significant (Figure 2).

In the test group, the mean PLQ value was 73% (SD: ±15.2), while in the control group, it was lower, at 24.4% (SD: ±9.4). The difference in PLQ between the test and control groups was statistically significant (*p* < 0.001).

The mean PPD value for the test group was 5.3 mm (SD: ±1.1), while for the control group, a lower mean value of 1.6 mm (SD: ±0.8) was recorded. The difference in PPD between the test group and the control group was statistically significant (*p* < 0.001).

The mean BPI was higher in the test group, at 82% (SD: ±10.5), compared to the control group, i.e., 23% (SD: ±8.2). The difference in BPI between the test group and the control group was statistically significant (*p* < 0.001).

The Pearson correlation analysis revealed that the only statistically significant strong positive correlation was between the educational status and the number of teeth in patients of the test group (Table 2). Other correlations between the educational status and periodontal parameters were weak and had no statistical significance (*p* > 0.05) (Table 3).

### 3.3. Impact of Dental Implants

The values of the assessed periodontal parameters decreased more in patients with hypertension and dental implants than in those without dental implants. Nevertheless, the differences remained statistically significant compared to control patients with dental implants (Table 4).

The mean PLQ for the test group was 24% (SD: ±5.2) compared to 15.6% for the control group (SD: ±3.8), with the difference in PLQ between the test group and the control group with implants being statistically significant (*p* < 0.05).

The PPD for patients with implants was 4.4 mm (SD: ±0.6), while for the control group, it was 2.9 mm (SD: ±0.5). The difference in PPD between the test group and the control group with implants was statistically significant (*p* < 0.05).

Regarding the mean BPI, the value for the test group was 44% (SD: ±8.2), while the mean BPI for the control group was 15.5% (SD: ±4.5). The difference in BPI between the test group and the control group with implants was statistically significant (*p* < 0.05).

### 3.4. Overview of Findings

Oral hygiene was better in the control group, as shown by lower PLQ values. The hypertensive group had worse periodontal status, with higher PPD and BPI values. In both groups, patients with implants showed improved periodontal indicators, with more significant improvements observed in the control group. Hypertensive patients with implants had better periodontal statuses than those without.

## 4. Discussion

The findings of this study highlight the significant impact of cardiovascular pathology on oral hygiene and periodontal status. Patients with high blood pressure exhibited poorer periodontal status indicators, including increased PLQ, PPD, and BPI as compared to healthy controls. These results are consistent with previous studies that have established a connection between CVDs and PD [2,3,30]. Mediated by local and systemic inflammation, this link involves periodontal bacteria that enter blood circulation and trigger the formation of atheromatous plaques [31]. Together with the upregulated levels of IL-6 and CRP found in patients with CVD and PD, this highlights the strong bi-directional connection between the two types of conditions [32].

The demographic analysis revealed a balanced distribution of educational and geographic factors between the test and control groups, minimizing potential biases in the results. Most participants in both groups were high-school graduates, with a smaller proportion having university degrees, reflecting a relatively homogeneous educational status across the study population. This distribution is important as educational status often influences health literacy, access to dental care, and adherence to oral hygiene practices [33]. Furthermore, for both groups, the correlations between the educational status and periodontal parameters were weak and had no statistical significance. This showed that the educational level of the patients had little impact on their periodontal status, influencing it to a lesser extent than the existence of elevated blood pressure.

Additionally, participants with dental implants originated from both rural and urban areas, which supports the representativeness of the study sample. However, the rural-to-urban ratio indicated a slight urban predominance in both groups, which may point to better accessibility of implant therapy in urban settings. This finding underscores the potential disparities in healthcare access between rural and urban populations, which could influence oral health outcomes. Despite these similarities, the hypertensive group exhibited significantly worse periodontal status, highlighting that systemic health conditions, rather than educational or geographic factors alone, play a critical role in periodontal status. These results emphasize the need for tailored public health interventions that consider both medical and educational factors to improve periodontal outcomes, particularly in vulnerable populations such as those with hypertension.

In our study, hypertensive patients exhibited significantly worse periodontal status, as indicated by higher PLQ, PPD, and BPI values. These findings are consistent with those of Franek et al., who reported that arterial hypertension is associated with increased periodontal inflammation and higher rates of periodontal disease progression [34]. The systemic inflammation related to hypertension may contribute to a more pronounced inflammatory response in the periodontal tissues, exacerbating the severity of PD [22].

Other research studies have also established a correlation between hypertension and increased periodontal disease. For instance, Holmlund et al. observed that patients with hypertension had significantly higher periodontal disease prevalence compared to normotensive individuals [35]. Furthermore, evidence suggests that managing periodontal disease can positively impact cardiovascular health. A study by D’Aiuto et al. demonstrated that intensive periodontal treatment reduced systemic inflammation and improved endothelial function in patients with both CVD and PD [32].

Good oral hygiene is crucial for preventing periodontal diseases by reducing plaque buildup, which is a key factor in the development of gingivitis and periodontitis [30,31]. In hypertensive patients, poor oral hygiene can exacerbate periodontal issues due to reduced immune function and increased systemic inflammation associated with hypertension. Data on plaque indices (PLQ) show that hypertensive patients tend to have worse oral hygiene, leading to higher plaque accumulation and a poorer periodontal status (e.g., higher PPD and BPI values) [35]. Maintaining proper oral hygiene can prevent plaque-related damage and improve periodontal health, especially in patients with hypertension, as seen in the control group with better plaque control and periodontal indicators [35].

Oral hygiene can be influenced by a patient’s perception of their disease and the medications they take. Hypertensive patients may not always prioritize oral care due to the lack of immediate symptoms of periodontal disease, leading to poorer hygiene practices. Medications like diuretics, commonly prescribed for hypertension, can cause xerostomia (dry mouth), which reduces saliva flow and impairs the mouth’s natural cleaning mechanism [19]. This results in increased plaque accumulation and a higher risk of periodontal issues. Xerostomia can also cause discomfort, further discouraging good oral hygiene practices, making regular brushing and flossing more challenging for these patients [20].

Function restoring and aesthetics in patients with tooth loss have become the standard therapeutical approach in modern day dentistry, as dental surgery has become more and more accessible to patients [36]. The outcomes of dental implant placement are favorable even for patients with diabetes, as long as good oral hygiene is kept and the implants are well maintained through regular recalls [37,38,39]. The insertion of dental implants can also have a positive impact on the periodontal status of patients, providing stable support for prosthetic teeth and facilitating improved oral hygiene practices [28]. In compliance with the results obtained throughout our study, both hypertensive and healthy patients with dental implants exhibited better periodontal status indicators compared to their counterparts without implants. Despite the reduced sample, these preliminary results encourage future developments on the subject, suggesting that dental implant therapy in patients with hypertension could contribute to a significant improvement in their oral health. Potentially, by enforcing good oral health hygiene practices and through constant follow-up visits, patients with dental implants could reach a stable and predictable periodontal status [38].

Interestingly, with the perspective of developing these preliminary results in the future, while the periodontal status improvements were more pronounced in the control group, hypertensive patients with implants also showed significant reductions in PLQ, PPD, and BPI. Our findings align with the research work performed by Pjetursson et al., who validated that dental implants can reduce the periodontal burden by redistributing occlusal forces and minimizing the impact on the remaining natural teeth [39]. Moreover, Salvi et al. found that peri-implant health was maintained in patients with well-controlled systemic conditions, including hypertension, suggesting that dental implants can be a viable option for patients with cardiovascular conditions [40].

The results of our study show significant outcomes in clinical practice, particularly in the management of periodontal conditions in patients with hypertension. Given the higher risk of periodontal disease in hypertensive patients, regular periodontal assessments and targeted interventions are crucial [41]. Dental implants may offer a valuable therapeutic option for improving oral hygiene and periodontal status in this high-risk population [27]. Moreover, the results highlight the importance of a multidisciplinary approach to patient care. Collaboration between cardiologists and dental professionals can facilitate the early identification and management of periodontal issues in patients with CVD, thus contributing to enhanced overall health outcomes. In the same vein, a recent study endorsed the idea that patients receiving comprehensive periodontal care exhibit a lower incidence of cardiovascular events [42].

Although our study provides valuable insights, it has several limitations. The small sample size of this study poses several limitations. The reduced number of participants may result in lower statistical significance, making it challenging to identify significant differences in periodontal status outcomes. Additionally, the sample is drawn from a specific region, potentially limiting the applicability of the results to broader or more diverse groups. Patients were selected only from one cardiology clinic in the region, generating specific addressability characteristics. Consequently, while the study provides important preliminary insights, the limitations highlight the need for larger-scale multi-centric research to validate these findings and explore the relationships between hypertension, oral hygiene, dental implants, and periodontal status more comprehensively. As described by the practical guidelines of periodontal therapy, good and constant patient education is key for optimal biofilm control in both patients with compromised periodontal status and in patients receiving dental implants [22,43].

Future longitudinal studies with larger sample sizes are needed to confirm these results and explore the long-term impact of dental implants on periodontal status in patients with CVD. Additionally, further research should investigate the underlying mechanisms linking CVD and PD, as well as the potential benefits of various periodontal therapies in this population. Understanding these mechanisms could lead to more effective strategies for managing periodontal conditions in patients with cardiovascular conditions [44]. Within the findings of our study, a recent review of former research evidence showed that periodontal treatment was associated with reduced systemic inflammation and improved cardiovascular outcomes [44]. The data suggest that while cardiovascular pathology negatively impacts oral hygiene and periodontal status, the insertion of dental implants can lead to significant improvements in these parameters. The improvements observed in hypertensive patients with implants underscore the potential benefits of dental implants in managing periodontal conditions in high-risk populations [45].

The findings highlight the importance of oral hygiene in managing periodontal health, especially for hypertensive patients who are at increased risk due to factors like medications and disease-related challenges. To improve dental care for these patients, an integrated approach between cardiologists and dentists is essential. Cardiologists should screen for oral health issues and provide guidance on managing medications that may contribute to dry mouth, while dentists should monitor and treat periodontal conditions in hypertensive patients.

Educational programs focused on proper oral hygiene practices, specifically adapted for hypertensive individuals, can help raise awareness about the link between oral health and overall well-being. Collaboration between healthcare providers can also ensure that both hypertension and periodontal health are managed simultaneously, with regular check-ups for early detection and intervention. This combined approach can improve both oral and systemic health outcomes for hypertensive patients. This approach was implemented at the end of our study, as participants were referred for oral hygiene guidance and periodontal treatment in the periodontology clinic of our university.

## 5. Conclusions

In conclusion, our study highlights that patients with high blood pressure have a less favorable periodontal status compared to healthy controls. However, the insertion of dental implants could improve periodontal status indicators in both hypertensive and healthy patients. These findings stress the need for early intervention and regular monitoring. An integrated approach between cardiologists and dentists, along with targeted education on oral hygiene, is crucial for improving both hypertension and periodontal health, leading to better patient outcomes.

## Figures and Tables

**Table 1:** Demographic data.

Group	Mean Age (Years)	Mean Teeth (n)/Patient	Mean Implants (n)/Patient	Gender Male/Female Ratio	Rural/Urban Ratio
Test	62.2	6.75	4.50	1.66	1.18
Control	47.7	17.03	4.00	1.72	1.63
*t*-test *p*-value	>0.05	<0.05	>0.05	>0.05	>0.05

**Table 2:** Correlation analysis between educational and periodontal status for test group.

Educational Level	PLQ	PPD	BPI	No. of Teeth
R	0.30	−0.03	0.22	0.76
*p*	0.145	0.86	0.30	0.00001

R = Pearson’s R; *p* = statistical significance (≤0.05).

**Table 3:** Correlation analysis between educational and periodontal status for control group.

Educational Level	PLQ	PPD	BPI	No. of Teeth
R	0.29	−0.07	0.11	0.23
*p*	0.11	0.9	0.55	0.20

R = Pearson’s R; *p* = statistical significance (≤0.05).

**Table 4:** Periodontal parameters for patients with dental implants.

Group	PLQ (%)	PPD (mm)	BPI (%)
Test	24	4.4	44
Control	15.6	2.9	15.5
ANOVA *p*-value	<0.05	<0.05	<0.05

## Data Availability

Data used to support the findings of this study are available from the corresponding author upon request. The data are not publicly available due to ethical considerations.
